# Allosteric effects of SSB C-terminal tail on assembly of *E. coli* RecOR proteins

**DOI:** 10.1093/nar/gkaa1291

**Published:** 2021-01-15

**Authors:** Min Kyung Shinn, Alexander G Kozlov, Timothy M Lohman

**Affiliations:** Department of Biochemistry and Molecular Biophysics, Washington University in St. Louis School of Medicine, St. Louis, MO 63110, USA; Department of Physics, Washington University in St. Louis, St. Louis, MO 63130, USA; Department of Biochemistry and Molecular Biophysics, Washington University in St. Louis School of Medicine, St. Louis, MO 63110, USA; Department of Biochemistry and Molecular Biophysics, Washington University in St. Louis School of Medicine, St. Louis, MO 63110, USA

## Abstract

*Escherichia coli* RecO is a recombination mediator protein that functions in the RecF pathway of homologous recombination, in concert with RecR, and interacts with *E. coli* single stranded (ss) DNA binding (SSB) protein via the last 9 amino acids of the C-terminal tails (SSB-Ct). Structures of the *E. coli* RecR and RecOR complexes are unavailable; however, crystal structures from other organisms show differences in RecR oligomeric state and RecO stoichiometry. We report analytical ultracentrifugation studies of *E. coli* RecR assembly and its interaction with RecO for a range of solution conditions using both sedimentation velocity and equilibrium approaches. We find that RecR exists in a pH-dependent dimer-tetramer equilibrium that explains the different assembly states reported in previous studies. RecO binds with positive cooperativity to a RecR tetramer, forming both RecR_4_O and RecR_4_O_2_ complexes. We find no evidence of a stable RecO complex with RecR dimers. However, binding of RecO to SSB-Ct peptides elicits an allosteric effect, eliminating the positive cooperativity and shifting the equilibrium to favor a RecR_4_O complex. These studies suggest a mechanism for how SSB binding to RecO influences the distribution of RecOR complexes to facilitate loading of RecA onto SSB coated ssDNA to initiate homologous recombination.

## INTRODUCTION

Recombination mediator proteins (RMPs) are essential factors in genome maintenance as they facilitate initiation of homologous recombination. Examples of RMPs include the UvsY protein of phage T4 (1–4), RecFOR proteins in prokaryotes (5–7), as well as Rad52 (8–13) and the Breast Cancer susceptibility 2 (BRCA2) proteins in eukaryotes (14–17). Single stranded DNA binding (SSB) proteins, such as T4 phage gp32 (18–20), bacterial SSB (21–28) and eukaryotic RPA (29–32), occupying damaged single-stranded DNA (ssDNA) must hand off the ssDNA to RMPs that then load recombinases (e.g. RecA (7,21) and Rad 51 (29,33)), onto SSB coated ssDNA, activating DNA repair. Mutations of human RMPs are implicated in several diseases including a predisposition to cancer and premature aging (34–38). However, these crucial interactions between SSBs and RMPs are poorly understood.

The RecF and RecBCD pathways are the two major pathways for DNA repair by homologous recombination in *Escherichia coli* (39–42). The RecF pathway primarily functions on single stranded DNA gaps whereas the RecBCD pathway repairs double stranded DNA breaks. However, the RecF pathway can also repair double stranded breaks when the RecBCD pathway is disabled (43,44). The RecF pathway has many recombination mediator protein components, including RecQ helicase, RecJ exonuclease, RecF, RecO, and RecR proteins (45–48).

**Figure 1.**
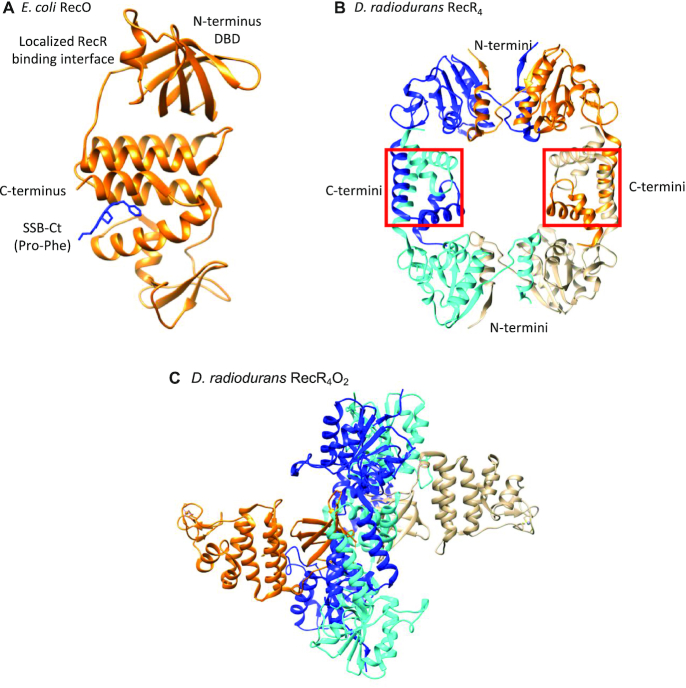
Structures of RecO and RecR. (**A**) Crystal structure of *E. coli* RecO monomer (orange ribbon) bound to a SSB C-terminal acidic tip peptide, WP9 (blue stick) (22,120). Only the last two C-terminal residues of the SSB tip, Pro and Phe, are observed bound to a hydrophobic pocket of the central alpha helical region of RecO. RecO is composed of the N-terminal DNA binding domain, central alpha helical region, and C-terminal zinc binding motif, although zinc is not observed in the *E. coli* structure. (**B**) Crystal structure of *D. radiodurans* RecR tetramer (87). Each RecR monomer is colored in blue, cyan, orange, and gold. *D. radiodurans* RecR assembles via its C-termini by swapping Walker B motifs and at the N-termini by swapping HhH motifs (87). (**C**) Crystal structure of ‘closed’ *D. radiodurans* RecR_4_O_2_ complex viewed from the side (90). Two RecO molecules (orange and gold) are bound on each side of the tetrameric ring of RecR (alternating cyan and blue for each subunit). The RecO-RecR interaction site is localized at the N-terminal DBD in RecO (90) and near the central hole in RecR_4_ ring. Each RecO is situated near the middle of a monomer subunit of a RecR_4_ ring and interacts with residues from both the N-terminal Walker B motifs and C-terminal HhH motifs, which are important in domain swapping to form a RecR tetramer. RecO is also in contact with residues in the C-terminal Toprim domain.

*Escherichia coli* RecO protein is composed of an N-terminal DNA binding domain (Figure 1A), which binds both ss and dsDNA and can facilitate the annealing of two complementary single strands of DNA (22,49,50). RecO overcomes the inhibitory effect of SSB bound to ssDNA to anneal complementary DNA strands (22,49,51). The C-terminal domain of RecO includes a central alpha helical region and a zinc-binding motif (22), although zinc is not observed in the *E. coli* RecO crystal structure. RecO is one of many SSB interacting proteins (SIPs) (26,28) that bind to the last nine amino acids (MDFDDDIPF) of the intrinsically disordered C-terminal tail of *E. coli* SSB (SSB-Ct), termed the acidic tip (22). This highly acidic tip interacts with SIPs with specificity (26,52). One of the essential roles that SSB plays in genome maintenance is to act as a hub to recruit more than 17 SIPs involved in recombination (22,45,51,53–62), replication (63–67), replication restart (68–71) and repair (72–80) via the tip (28). As SSB is a homo-tetramer (81,82), up to four SIPs can potentially bind per SSB tetramer (26). The last two residues of the tip (Pro and Phe) are observed bound to a hydrophobic pocket in the central alpha helical region of RecO in a crystal structure (Figure 1a) (22).

RecO alone has ssDNA binding and DNA annealing activity (22,49), but requires RecR to stimulate RecA loading (21,60,83). *Escherichia coli* RecR does not bind SSB or DNA (60,83), unlike *Deinococcus radiodurans* RecR which has a ring-like structure (Figure 1B) and binds both ssDNA and double-stranded DNA (dsDNA) (84). In addition to the tetrameric structure observed in crystal structures, single-molecule experiments have shown that *D. radiodurans* RecR also forms stable dimers via an N-terminal interaction (85). No crystal structures are available for *E. coli* RecR, however, sucrose gradient sedimentation studies suggested that *E. coli* RecR forms a dimer (60). A dimeric model of *Thermus thermophilus* RecR has also been proposed based on an NMR study (86). Furthermore, like the crystal structures of RecR from *D. radiodurans* (87), *Pseudomonas aeruginosa* (88) and *Thermoanaerobacter tengcongensis* (89) show ring-like tetrameric structures, and structures of *D. radiodurans* RecR show an additional dimer of concatenated tetramers suggesting that the ring can open and close (87). Whereas crystal structures of *D. radiodurans* RecOR complex show a stoichiometry of two RecO molecules bound to a RecR tetramer (Figure 1C), it has been suggested that an *E. coli* RecOR complex exists as a RecR_2_O_2_ complex (84,87,90–93). Hence, the assembly states of *E. coli* RecR and its complexes with RecO are not well defined.

In order to clarify this, we investigated the assembly states and thermodynamics of assembly of *E. coli* RecR and RecO by analytical ultracentrifugation. We also investigated the equilibrium binding of *E. coli* RecO to RecR to determine the assembly state of RecR to which RecO binds and examined the effect of the SSB C-terminal acidic tip on the energetics of the RecO–RecR interaction.

## MATERIALS AND METHODS

### Buffers and reagents

Buffers were prepared with reagent grade chemicals using distilled, deionized water (Milli-Q system; Millipore corp., Bedford, MA, USA). Spectrophotometric grade glycerol was from Alfa Aesar (Ward Hill, MA, USA). Buffer BTP is 20 mM Bis-Tris Propane (pH 8.0 at 25°C), 50 mM NaCl, 25% (v/v) glycerol, 1 mM TCEP. Any variations in pH, temperature, NaCl or MgCl_2_ concentration are indicated in the text.

### Proteins, peptides, and DNA

*Escherichia coli* RecO protein was overexpressed from plasmid pMCSG7 in *E. coli* strain BL21(DE3)pLysS (kindly provided by Dr Sergey Korolev, Saint Louis University) and purified using Ni-NTA affinity chromatography and a HiTrap Heparin HP affinity column (GE Healthcare, Chicago, IL, USA) after His-tag cleavage with TEV protease as described (22). The auto-inactivation-resistant S219V mutant of TEV protease with an N-terminal His-tag and C-terminal polyarginine tag (His-TEV(S219V)-Arg) was overexpressed from *E. coli* strain BL21(DE3) transformed with PRK793 and pRIL (Stratagene, San Diego, CA, USA) and purified as described (94). *Escherichia coli* RecR protein was overexpressed from plasmid pMCSG7 in *E. coli* strain BL21 Rosetta 2(DE3)pLysS (kindly provided by Dr Sergey Korolev) and purified using Ni-NTA affinity chromatography, followed by cleavage of the His-tag with TEV protease as described (92). The concentrations of RecO and RecR in monomers were determined in buffer BTP from their absorption spectra using extinction coefficients of ϵ_280_ = 2.44 × 10^4^ and 5.96 × 10^3^ M^−1^cm^−1^, respectively, as determined from their amino acid sequences by SEDNTERP (95).

A peptide containing the 15 C-terminal amino acids (PSNEPPMDFDDDIPF) of *E. coli* SSB, denoted P15, was purchased from WatsonBio (Houston, TX, USA). The P15 peptide concentration was determined in buffer BTP from its absorption spectrum using an extinction coefficient of ϵ_258_ = 390 M^−1^ cm^−1^ due to its two Phe residues.

### Analytical ultracentrifugation (AUC)

Sedimentation velocity and equilibrium experiments were performed with an Optima XL-A analytical ultracentrifuge using An50Ti or An60Ti rotors (Beckman Coulter, Fullerton, CA, USA) at 42 000 rpm for sedimentation velocity and at the indicated rotor speeds between 18,000 rpm and 30 000 rpm for sedimentation equilibrium experiments at 25°C as described (96). Absorbance was monitored at 230 nm for all experiments except for sedimentation velocity experiments of RecR which were monitored at 233 nm.

The densities and viscosities of the buffers at 25°C were calculated using SEDNTERP (95). The partial specific volumes, $\bar{\upsilon }$, of RecO and RecR were determined experimentally from independent sedimentation equilibrium experiments on the individual proteins in buffer BTP. The molecular weights of RecO and RecR were constrained to their known values based on their amino acid sequence and the value of $\bar{\upsilon }$ was obtained by floating $\bar{\upsilon }$ in a non-linear least squares (NLLS) analysis of the SE data. The values of $\bar{\upsilon }$ determined in buffer BTP are 0.734 ml/g for RecO and 0.711 ml/g for RecR. These differ from the values calculated using SEDNTERP (0.743 ml/g for RecO and 0.731 ml/g for RecR). The $\bar{\upsilon }$ of the P15 peptide was calculated using SEDNTERP, yielding 0.704 ml/g. In experiments involving more than one species, the partial specific volumes of the complexes were calculated assuming additivity using Equation (1), where *n_i_* = number of moles of species ‘*i*’, *M_i_* = molecular weight of species ‘*i*’, and $\overline {{\upsilon _i}}$ = partial specific volume of each species ‘*i*’.

(1) $$\begin{equation*}\bar{\upsilon } = \frac{{\mathop \sum \nolimits_i {n_i}{M_i}\overline {{\upsilon _i}} }}{{\mathop \sum \nolimits_i {n_i}{M_i}}}\end{equation*}$$

The calculated values of $\bar{\upsilon }$ for the RecR_4_O and RecR_4_O_2_ complexes in buffer BTP are 0.716 and 0.720 ml/g, respectively.

### Sedimentation velocity data

Sample (380 μl) and buffer (394 μl) were loaded into each sector of an Epon charcoal-filled two-sector centerpiece. Absorbance data were collected by scanning the sample cells at intervals of 0.003 cm. Data were analyzed using Sedfit to obtain *c*(*s*) distributions (97). The *c*(*s*) distribution function defines the populations of species with different sedimentation rates and represents a variant of the distribution of Lamm equation solutions (97). Weight average sedimentation coefficients were obtained by integrating the *c*(*s*) distributions over the entire sedimentation coefficient range used for fitting the data in Sedfit (95).

Sedimentation velocity experiments were performed at multiple RecO (ranging from 1.5 to 8 μM) and RecR concentrations (ranging from 2 to 20 μM monomer) as indicated in the text and figure legends. In titrations of RecO by RecR, 1.5 μM RecO was titrated by increasing concentrations of RecR (1.5–9 μM) at [RecO]:[RecR] molar ratio of 1:1, 1:2, 1:3, 1:4 and 1:6. In forward titrations of RecR by RecO, 2 μM of RecR was titrated by 4 and 6 μM of RecO at [RecO]:[RecR] molar ratio of 2:1 and 3:1.

### Sedimentation velocity simulations

**Scheme 1.**
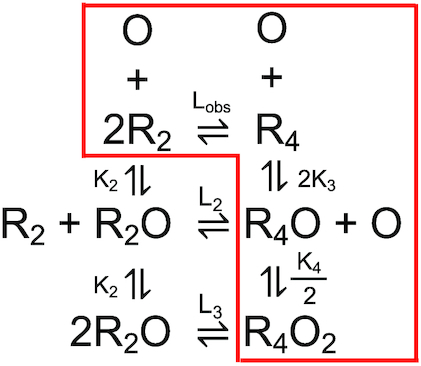
Two molecules of RecO binding to RecR in a dimer–tetramer equilibrium. Only the species in the red box are observed at equilibrium.

The program SedAnal (98) was used to simulate the sedimentation velocity experiments performed with RecO and RecR shown in Figure 4A and B (buffer BTP (pH 8.0) at 25°C) using the model in Scheme 1 that describes the association of RecO with RecR that exists in a dimer-tetramer equilibrium. *L*_obs_ is the RecR tetramerization equilibrium constant. *K*_2_, *K*_3_ and *K*_4_ are the step-wise association equilibrium constants, corrected for statistical factors, for RecO binding to RecR_2_, R_4_ and R_4_O, respectively. Simulations were performed for RecO (1.5 μM) and RecR (1.5–9 μM) at [RecO]:[RecR] molar ratio of 1:1, 1:2, 1:3, 1:4 and 1:6 as in experiments in Figure 4A and B for a range of *K*_3_ and *K*_4_ values. The value of *L*_obs_ was fixed as this was obtained from analysis of an independent set of sedimentation equilibrium experiments with RecR alone (Figure 3 biv, (2.16 ± 0.05) × 10^5^ M^−1^). *K*_2_ was fixed at a low value (10 M^−1^) as the RecR_2_O species is not observed in any experiments (see Results). The reverse rate constants for all reactions were set to 0.01 s^−1^; lower values did not affect the results. The extinction coefficients at 230 nm were determined as 1.55 × 10^5^ M^−1^ cm^−1^ for RecO and 5.3 × 10^4^ M^−1^ cm^−1^ for RecR from known protein concentrations and extinction coefficients at 280 nm. The meniscus and the bottom of the cell were set at 6.14 cm and 7.2 cm, respectively. These values are similar to what we observe in typical sedimentation velocity experiments. Standard deviation of noise of 0.005 was added. The simulated data were then analyzed using Sedfit to obtain *c*(*s*) distributions (95).

### Sedimentation equilibrium data

Sedimentation equilibrium experiments with RecR were analyzed in two ways. In the first analysis, we determined that the two *c*(*s*) peaks observed by sedimentation velocity in Figure 3A correspond to RecR dimer and tetramer indicating that RecR exists in a dimer-tetramer equilibrium. This was done by constraining one species to have the molecular weight (MW) of a RecR dimer and floating the MW of the second species as described below. In the second analysis, we constrained the MW of the two species to be that of RecR dimer and RecR tetramer and then determined the dimer/tetramer equilibrium constant, *L*_obs_, from NLLS analysis as described below.

Sedimentation equilibrium experiments were performed at multiple RecR concentrations (ranging from 4 to 12 μM) and multiple rotor speeds (ranging from 20 000 to 30 000 rpm) as indicated in the text and figure legends. The protein sample (110 μl) and buffer (120 μl) were loaded into each sector of an Epon charcoal-filled six-channel centerpiece. Absorbance data were collected by scanning the sample cells at intervals of 0.003 cm in the step mode with 5 averages per step. Samples were sedimented to equilibrium at the indicated rotor speeds, starting with the lowest speed. Equilibrium was deemed to have been reached when successive scans made several hours apart showed no differences. The resulting absorbance profiles, *A*_r_, were analyzed using NLLS fitting to Equation (2) as implemented in Sedphat (99) to estimate molecular weights of the protein species using ‘Species Analysis with Mass Conservation Constraints’ model:

(2) $$\begin{eqnarray*} {A_r} &=& \mathop \sum \limits_{i = 1}^n {A_{{r_0},i}} \nonumber \\ && \times\, {\rm{exp}}\left[ {{M_i}\left( {1 - {{\bar{\upsilon }}_i}\rho } \right)\frac{{{\omega ^2}}}{{2RT}}\left( {{r^2} - r_0^2} \right)} \right] + {b_r} \end{eqnarray*}$$

where *r* is the distance from the center of rotation, *r_0_* is an arbitrary reference radius, ω is angular velocity, *T* is absolute temperature, *R* is the gas constant, *M_i_* is the molecular weight of species ‘*i*’, $\overline {{\upsilon _i}}$ = partial specific volume of each species ‘*i*’, ρ is the buffer density, ${A_{{r_0},i}}$ is the absorbance of species ‘*i*’ at the reference position, and *b_r_* is a radial-dependent baseline offset. For RecO experiments, the data were fit to a one species model. For RecR experiments, the data were fit to a two species model where the dimer molecular weight was constrained at 43.9 kDa and the tetramer molecular weight was floated.

To determine the tetramerization equilibrium constants, *L*_obs_ = [R_4_]/[R_2_]^2^, the RecR sedimentation equilibrium data were fit to a ‘Dimer–Tetramer’ equilibrium model (100):

(3) $$\begin{eqnarray*} {C_{{\rm total}}} &=& {C_{di,{r_{\rm o}}}}\ \exp\left[ {\frac{{{M_{di}}{\omega ^2}}}{{2RT}}\left( {1 - \bar{\upsilon }\rho } \right)\left( {{r^2} - r_{\rm o}^2} \right){\rm{ }}} \right] \nonumber \\ && + {L_{{\rm obs}}}{({C_{di,{r_{\rm o}}}})^2}\exp\left[ {\frac{{2{M_{di}}{\omega ^2}}}{{2RT}}\left( {1 - \bar{\upsilon }\rho } \right)\left( {{r^2} - r_{\rm o}^2} \right){\rm{ }}} \right] \nonumber \\ \end{eqnarray*}$$

where *C* is the concentration of denoted species and *C_di,ro_* is the concentration of dimer at the reference radius. The molecular weight of the dimer, *M_di_*, was constrained at 43.9 kDa and *L*_obs_ was determined by NLLS analysis using Equation (3).

Species fractions of RecR were simulated using the tetramerization equilibrium constants obtained from sedimentation equilibrium experiments using Scientist (MicroMath Scientist Software, St. Louis, MO) using Equations (4) and (5),

(4) $$\begin{equation*}{L_{{\rm obs}}} = \frac{{\left[ {{{\rm R}_4}} \right]}}{{{{[{{\rm R}_2}]}^2}}}\end{equation*}$$

(5) $$\begin{equation*}[{{\rm R}_{{\rm tot}}}]\ = \ 2{L_{{\rm obs}}}{\left[ {{{\rm R}_2}} \right]^2} + \left[ {{{\rm R}_2}} \right]\end{equation*}$$

where the fractions of dimer and tetramer are [R_2_]/[R_tot_] and [R_4_]/[R_tot_], respectively, and *R*_tot_ is the RecR concentration in dimer units.

The tetramerization equilibrium constants obtained were plotted in Figure 3D as log(*L*_obs_) as a function of pH and were fit to a model of *m* protonation sites on a tetramer and *n* protonation sites on a RecR dimer as described by Equation (6),

(6) $$\begin{equation*}{L_{{\rm obs}}} = {L_0}\ \frac{{{P_{{\rm tet}}}}}{{{{({P_{{\rm di}}})}^2}}}\end{equation*}$$

where *L*_0_ is the tetramerization equilibrium constant in the absence of protonation, and *P*_tet_ and *P*_di_ are the binding polynomials describing protonation of R_4_ and R_2_, respectively. The binding polynomials are *P*_tet_ = (1 + *k*_tet_[H^+^])^*m*^ and *P*_di_ = (1 + *k*_di_[H^+^])^*n*^ for *m* and *n* identical and independent protonation sites, and the binding polynomial for *m* cooperative protonation sites on R_4_ is *P*_tet,coop_ = 1 + (*k*_tet_[H^+^])^*m*^. The *k*_tet_ and *k*_di_ are the equilibrium constants for protonation of the RecR tetramer and dimer, respectively. The binding polynomials *P*_tet,coop_ and *P*_di_ were used to fit the data in Figure 3d and Supplementary Figure S1b using Equation (7). *P*_tet_ and *P*_di_ were used to fit the data in Supplementary Figure S1a.

(7) $$\begin{eqnarray*} \log \left( {{K_{{\rm obs}}}} \right) &=& \log \left( {{K_0}} \right)\ + \log \left( {1 + {{({k_{{\rm tet}}}{[H^ +] })}^m}} \right) \nonumber \\ && - 2n\ {\rm{log}}\left( {1 + {k_{{\rm di}}}{[H^ +] }} \right) \end{eqnarray*}$$

Sedimentation equilibrium experiments were performed at two different molar ratios of RecO (1.5 μM) to RecR (1.5 and 6 μM in monomers) and multiple rotor speeds (ranging from 18 000 to 29 000 rpm) as indicated in the text and figure legends.

Buffer BTP used in our experiments contains 25% (v/v) glycerol, which increases the solubility of the proteins and thus concerns can be raised about whether a glycerol gradient that can form may affect the conclusions drawn from the sedimentation experiments. Although a glycerol gradient will form in these experiments, its effect does not influence our conclusions as discussed in Supplementary Materials.

### Isothermal titration calorimetry (ITC)

Isothermal titration calorimetry (ITC) experiments were performed using a VP-ITC titration microcalorimeter (Malvern Panalytical, Malvern, UK) (101). All proteins and peptides were dialyzed extensively against the indicated buffer and cleared by centrifugation at 14 000 rpm for 15 min at 4°C after which the protein concentrations were determined. P15 (50 μM) was titrated into a pre-mixed solution of RecO (2 μM) and RecR (8 μM_monomer_) in buffer BTP, pH 8.0 at 25°C. The heats of dilution were obtained by blank titrations in which P15 was titrated into a solution containing only RecR (8 μM_monomer_), and corrections for heats of dilution were applied.

The raw data were analyzed to obtain titration curves by integrating each peak from the time of titrant addition until equilibration back to the baseline using ‘MicroCal Data Analysis’ software provided by the manufacturer. The binding parameters, stoichiometry (*n*), observed association equilibrium constant (*K*_obs_) and binding enthalpy (Δ*H*_obs_), were obtained by fitting the titration curves to a model of P15 (*X*) binding to *n* identical and independent sites on the RecO (*M*) using Equation (8),

(8) $$\begin{equation*}Q_i^{{\rm tot}} = {V_0}\ \Delta {H_{{\rm obs}}}{M_{{\rm tot}}}\frac{{n{K_{obs}}X}}{{1 + {K_{{\rm obs}}}X}}\end{equation*}$$

where $Q_i^{{\rm tot}}$ is the total heat after the *i*th injection and ${V_0}$ is the volume of the calorimetric cell. The concentration of the free ligand (*X*) was obtained by solving Equation (9):

(9) $$\begin{equation*}{X_{{\rm tot}}} = \ X + \ {X_{{\rm bound}}} = \ X + \frac{{n{K_{{\rm obs}}}x\ }}{{1 + {K_{{\rm obs}}}x\ }}{M_{tot}}\end{equation*}$$

In Equations (8) and (9), ${X_{{\rm tot}}}$ and ${M_{{\rm tot}}}$ are the total concentrations of P15 and RecO, respectively, in the calorimetric cell after ‘*i’*th injection and *x* is the free P15 concentration. Nonlinear least-squares fitting of the data was performed using the MicroCal Data Analysis software. The conversion of integral heats ($Q_i^{{\rm tot}}$) to differential heats (heats per injection observed in the experiment) and the fitting routine including corrections for heat displacement effects and ligand and macromolecule dilutions in the calorimetric cell were performed as described (102).

## RESULTS

### *E. coli* RecO is monomeric

**Figure 2.**
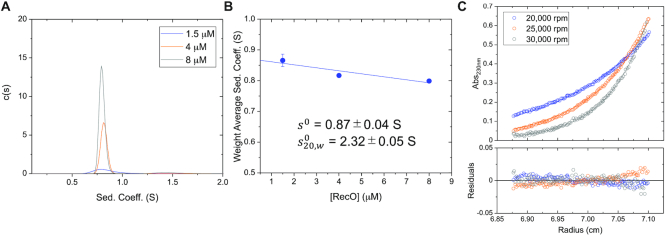
*E. coli* RecO is monomeric. (**A**) Sedimentation velocity c(s) distribution profiles of RecO (monitored at 230 nm) at 1.5, 4 and 8 μM in buffer BTP (pH 8.0) at 25°C. (**B**) The weight average sedimentation coefficient of RecO slightly decreases as a function of RecO concentration, expected for a pure, non-associating species (103–106). A linear extrapolation to zero RecO concentration yields *s*^0^ = 0.87 ± 0.04 S. From this we calculate $s_{20,w}^0$ = 2.32 ± 0.05 S, consistent with a RecO monomer. (**C**) Results of sedimentation equilibrium experiments (monitored at 230 nm) performed at 4 μM RecO at three rotor speeds 20,000 (blue), 25 000 (orange) and 30 000 (gray) rpm are shown in blue, orange and gray. Each equilibrium profile is described by a single exponential. The solid lines show the best global NLLS fit of the three data sets to one-species model with mass constraints (Equation (2)) (99) yields a molecular weight estimate of 25.3 ± 0.3 kDa, consistent with the predicted molecular weight of 27.4 kDa for a RecO monomer. The bottom panel shows the residuals, indicating good fits.

Previous studies using sucrose gradient sedimentation (60) and X-ray crystallography (22) indicate that RecO is a stable monomer (27.4 kDa) (5). To examine this further under the conditions of our experiments, we performed sedimentation velocity experiments in buffer BTP (pH 8.0) at 25°C at RecO concentrations from 1.5 to 8 μM. A c(s) distribution analysis (95) shows a single symmetric peak with sedimentation coefficient of ∼0.8 S (Figure 2A). The weight average sedimentation coefficient decreases slightly with increasing [RecO] (Figure 2B) as expected for a non-associating protein (103–106). A linear extrapolation to zero RecO concentration yields *s*^0^ = 0.87 ± 0.04 S in buffer BTP at 25°C. From this, we calculate $s_{20,w}^0$ = 2.32 ± 0.05 S, which is consistent with the $s_{20,w}^0$ = 2.47 S for a RecO monomer as estimated using WinHydroPRO (107) and a crystal structure of RecO (22,107).

Sedimentation equilibrium experiments with RecO (4 μM) in buffer BTP (pH 8.0) at 25°C were performed at three rotor speeds to determine an absolute molecular weight (108). Each equilibrium concentration profile (Figure 2C) was well described by a single exponential indicating a single species, and a global non-linear least squares (NLLS) fit of the three data sets to Equation (2) yields a molecular weight of 25.3 ± 0.3 kDa, consistent with the molecular weight of a RecO monomer (27.4 kDa) as calculated from its amino acid sequence (5). Hence, RecO is a stable monomer in buffer BTP at 25°C up to at least 8 μM.

### *E. coli* RecR exists in a dimer-tetramer equilibrium that is pH-dependent

**Figure 3.**
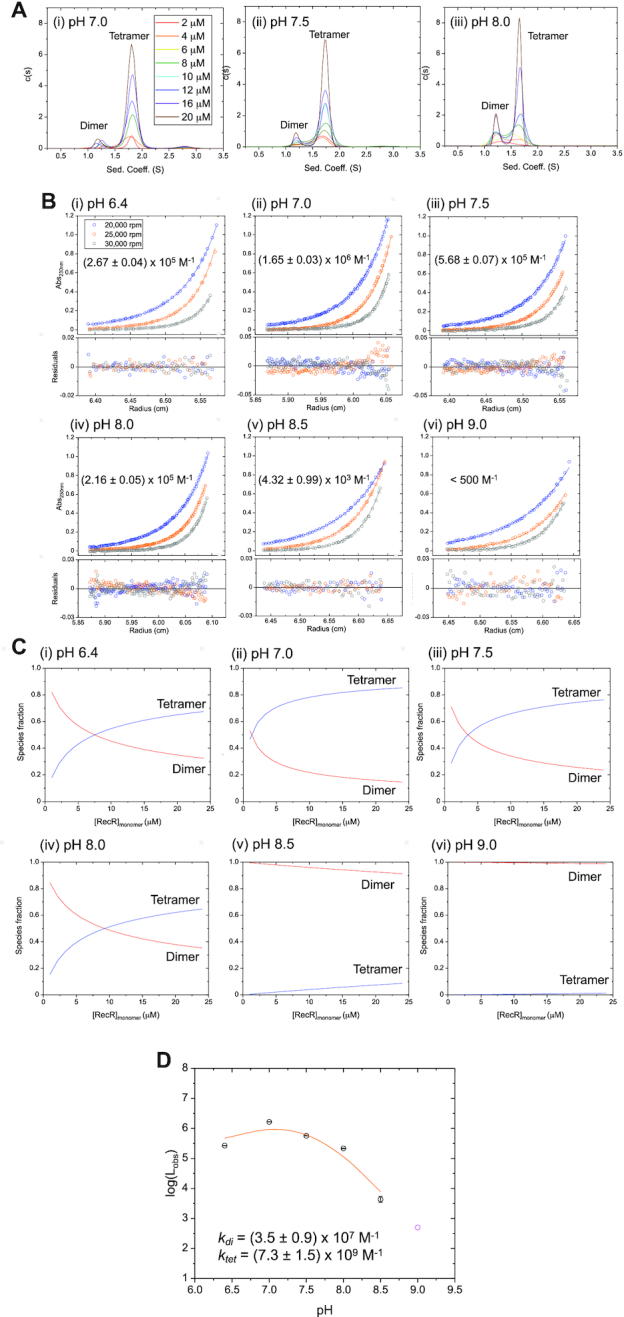
*E. coli* RecR exists in a pH-dependent dimer-tetramer equilibrium. (**A**) Sedimentation velocity (monitored at 233 nm) c(s) distribution profiles of 8 RecR concentrations between 2 and 20 μM (monomers) in buffer BTP at 25°C at (i) pH 7.0, (ii) pH 7.5, (iii) pH 8.0. Two major peaks are observed at 1.2 and 1.7 S, indicating two different assembly states of RecR. The area of the peak at 1.7 S increases with increasing RecR concentration. (**B**) Results of sedimentation equilibrium experiments (monitored at 230 nm) for RecR at 8 μM_monomer_ at 20 000 rpm (blue), 25 000 rpm (orange) and 30 000 rpm (gray) in buffer BTP at 25°C at (i) pH 6.4, (ii) 7.0 and (iii) pH 7.5 (iv) pH 8.0, (v) 8.5, (vi) 9.0. The solid lines show the best global NLLS fits of the three sets of data to a dimer-tetramer equilibrium model (Equation (3)) with MW of the RecR dimer and tetramer fixed at 43.9 and 87.8 kDa, respectively (99). The dimer-tetramer equilibrium constants, *L*_obs_ (noted in each panel and given in Table 1), were determined from this analysis. The residuals from the fits are shown in bottom panels. The data at pH 8.0 was also fitted to a two-species model with mass constraint where the MW of the RecR dimer was fixed at 43.9 kDa while the MW of the tetramer was fitted to 85.8 ± 0.2 kDa, close to the expected MW for a RecR tetramer (87.8 kDa). (**C**) Simulated species fractions of dimers (red) and tetramers (blue) plotted as a function of [RecR] (monomers). Fractions of each species were simulated with a dimer–tetramer model (Equations (4) and (5)) using the tetramerization equilibrium constants (L_obs_) obtained from sedimentation equilibrium experiments shown in panel (b) as a fixed parameter. Consistent with the sedimentation velocity c(s) profiles panel (a), the tetrameric population increases with increasing [RecR] and decreasing pH. (**D**) Plot of log(*L*_obs_) versus pH with obtained *L*_obs_ values from sedimentation equilibrium experiments in panel (B). The value of *L*_obs_ at pH 9.0 (magenta circle) is only an upper limit and was excluded from the following analysis. The solid line shows the best fit to a dimer–tetramer equilibrium model (Equation (7)) assuming two independent and identical protonation sites on the RecR dimer and three cooperative sites on the tetramer (*k*_tet_ = (7.3 ± 1.5) × 10^9^ M^−1^, *k*_di_ = (3.5 ± 0.9) × 10^7^ M^−1^). Other models of different number of protonation sites were considered but did not fit the data as well (Supplementary Figure S1).

We next examined the self-assembly of *E. coli* RecR protein using sedimentation velocity in buffer BTP (pH 8.0) at 25°C at eight concentrations between 2 – 20 μM (monomer). At each protein concentration, *c*(*s*) analysis indicates the presence of two major peaks at ∼1.2 and ∼1.7 S (Figure 3Aiii). The positions of the peaks do not change with RecR concentration suggesting that each peak represents a unique RecR species differing in assembly state. Upon increasing RecR concentration, the relative area of the peak at ∼1.7 S increases, while the relative area of the peak at ∼1.2 S decreases (Figure 3Aiii) indicating that these two RecR species exist in a slow equilibrium on the sedimentation time scale. The calculated *s*_20,w_ values of 2.0 and 2.9 are consistent with the two species representing RecR dimers and tetramers (109). This was verified by the sedimentation equilibrium analysis presented below.

We next performed sedimentation equilibrium experiments at 8 μM RecR (monomer) in buffer BTP (pH 8.0) at 25°C at three rotor speeds (Figure 3Biv), and the results were globally fit to a two-species model with mass constraint with one species fixed to the molecular weight (MW) of a RecR dimer (43.9 kDa) while allowing the MW of the second species to float. The resulting fit yielded a MW estimate of the second species as 85.8 ± 0.2 kDa, close to the expected molecular weight for a RecR tetramer (87.8 kDa). Based on this, we assign the two species observed in the sedimentation velocity experiments (Figure 3Aiii) to be RecR dimer (∼1.2 S) and RecR tetramer (∼1.7 S).

Sedimentation velocity c(s) profiles at eight RecR concentrations at pH 7.0 and 7.5 similarly show two major peaks at ∼1.2 and ∼1.7 S (Figure 3Ai and ii). A third very minor species (<2%) at ∼2.7 S is observed at pH 7.0 and 7.5, but not at 8.0. This species was not considered further in our analysis due to its low population. At each pH, increasing RecR concentrations increases the population of tetramers, however, for a given RecR concentration, the fraction of tetramers decreases with increasing pH.

We next performed sedimentation equilibrium experiments with RecR in buffer BTP at 25°C at concentrations of 4, 8 and 12 μM (monomer) at six pH values between 6.4 and 9 (Figure 3B and Supplementary Figure S3). We analyzed these data to obtain estimates of the dimer–tetramer equilibrium constants, *L*_obs_ = [R_4_]/[R_2_]^2^, at each pH by constraining the MW of the dimer and tetramer to their known values while fitting for *L*_obs_ using Equation (3). The global NLLS fits of the three concentrations at three rotor speeds for each pH are presented in Supplementary Figure S3 with a table of RMSD values for the fits in Supplementary Table S1. At pH 8.0, *L*_obs_ = (2.16 ± 0.05) × 10^5^ M^−1^. The values of *L*_obs_ obtained at pH 6.4, 7.0, 7.5, 8.0, 8.5 and 9.0 are given in Table 1 and plotted as log(*L*_obs_) versus pH in Figure 3D. Fractions of dimer and tetramer species at each pH were calculated using Equations (4) and (5) using *L*_obs_ obtained from sedimentation equilibrium experiments in Figure 3B and plotted against the concentration of RecR in monomers (Figure 3C). As observed in the sedimentation velocity experiments (Figure 3A), higher concentrations of RecR promote formation of the tetramer (Figure 3C, blue lines). RecR dimers are favored at high pH such that RecR is almost entirely dimeric at pH 9.0. In fact, we can only estimate an upper limit for *L*_obs_ < 500 M^−1^ at pH 9.

**Table 1.** Tetramerization equilibrium constants of RecR at various solution conditions. RecR dimer/tetramer equilibrium constants (25.0°C) were determined from sedimentation equilibrium experiments in buffer BTP and varying pH and salt concentrations as shown in Figure 3b and Supplementary Figure S2.

	Tetramerization equilibrium constant, *L*_obs_ (M^-1^)		
pH	50 mM NaCl	200 mM NaCl	10 mM MgCl_2_
6.4	(2.67 ± 0.04) × 10^5^		
7.0	(1.65 ± 0.03) × 10^6^		
7.5	(5.68 ± 0.07) × 10^5^		
8.0	(2.16 ± 0.05) × 10^5^	(3.49 ± 0.48) × 10^4^	(7.20 ± 0.83) × 10^4^
8.5	(4.32 ± 0.99) × 10^3^		
9.0	< 500		

The values of *L*_obs_ are nearly constant at low pH < 7.5, but then decrease steeply at pH > 7.5. The slope of the curve in Figure 3D at any point, ($\partial$log *L*_obs_/$\partial$pH) = Δ${n_{{{\rm H}^ + }}}$, where Δ${n_{{{\rm H}^ + }}}$ is the net difference in the number of protons taken up or released upon forming a tetramer from two dimers. At high pH (8.0–9.0), ($\partial$log *L*_obs_/$\partial$pH) = –2.5 ± 0.3, indicating a net uptake of two to three protons upon formation of the tetramer in this pH range. The dependence of *L*_obs_ on pH can be described by a model (Equation (7)) in which there are two independent and identical protonation sites on the RecR dimer and three cooperative protonation sites on a tetramer as described in Materials and Methods. The best fit values of the protonation equilibrium constants for the RecR dimer and tetramer, *k*_di_ and *k*_tet_, obtained from a NLLS fit of the data in Figure 3D to Equation (7) are (3.5 ± 0.9) × 10^7^ and (7.3 ± 1.5) × 10^9^ M^−1^. Other models were also considered (Supplementary Figure S1), but did not fit the data as well.

Addition of 10 mM MgCl_2_ to buffer BTP + 50 mM NaCl decreases *L*_obs_ to (7.20 ± 0.83) × 10^4^ M^−1^ (Supplementary Figure S2a) resulting in destabilization of the tetramer. Similarly, increasing [NaCl] to 200 mM decreases *L*_obs_ = (3.49 ± 0.48) × 10^4^ M^−1^ (Supplementary Figure S2b), thus decreasing tetramer stability. Experiments performed at 37°C in buffer BTP yield *L*_obs_ = (1.49 ± 0.17) × 10^5^ M^−1^ (Supplementary Figure S2c), similar to that estimated at 25°C [(2.16 ± 0.05) × 10^5^ M^−1^], indicating that the dimer-tetramer equilibrium is not affected much by temperature in this range.

### RecO binds to a RecR tetramer

**Figure 4.**
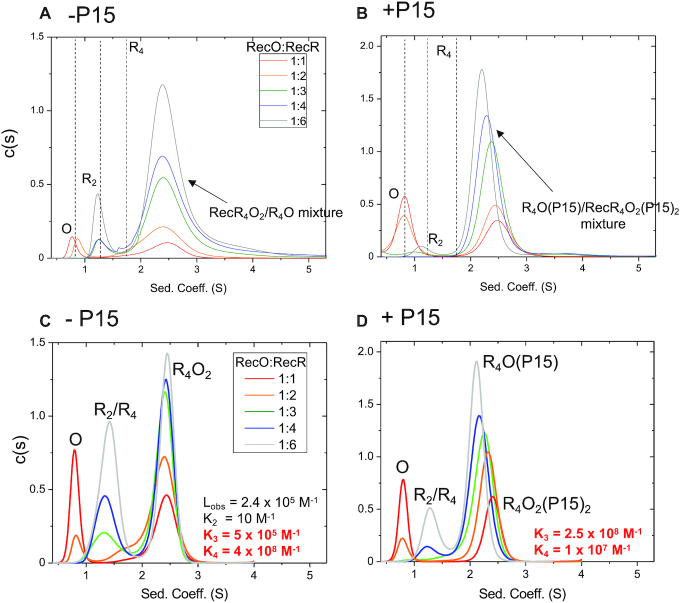
RecO binds to the RecR tetramer. RecO (1.5 μM) was titrated with increasing concentrations of RecR (1.5–9 μM) by sedimentation velocity (monitored at 230 nm) in the presence and absence of SSB-Ct peptide (P15) in buffer BTP (pH 8.0) at 25.0°C. (**A**) *c*(*s*) distribution profiles in the absence of P15 at the indicated molar ratios of [RecO]:[RecR] (monomers). The dotted lines show sedimentation coefficients for individual RecO, RecR_2_ and RecR_4_ species determined from isolated proteins. At 1:1 (red) and 1:2 (orange), the distributions show free RecO species at 0.8 S and a RecOR complex at 2.5 S. At molar ratios of 1:3 (green), 1:4 (blue), 1:6 (gray), free RecR dimer is observed at 1.2 S in addition to RecOR complex peaks at ∼2.5 S that show a slight shift to the left with increasing [RecR]. Since free RecO is not observed at the molar ratio of 1:3 and beyond, the observation of free RecR_2_ peak with RecOR peaks that shift to a lower sedimentation coefficient value indicate that a mixture of RecOR complex is present and that a RecOR complex with a lower MW forms at higher [RecR]. (**B**) In the presence of P15, free RecO is observed at 0.8 S for 1:1 (red) and 1:2 (orange) molar ratios in addition to a RecOR complex peak at ∼2.5 S. A free RecR_2_ species (1.2 S) is observed at higher [RecR]. Note that the RecOR complex peaks notably shift from 2.5 to 2.3 S at higher [RecR], indicating a significant formation of RecOR complex of lower MW. This shift is notably more significant than in the absence of P15. Panels (c) and (d) show simulations of titration of RecO (1.5 μM) with RecR (1.5–9 μM) by sedimentation velocity experiments monitoring at 230 nm at 42 000 rpm in buffer BTP (pH 8.0) at 25°C. (**C**) c(s) distribution profiles of simulated data with *K*_3_ = 5 × 10^5^ and *K*_4_ = 4 × 10^8^ M^−1^ describe the experimental data best in the absence of P15. A free RecO species is observed at 0.8 S, free RecR species at ∼1.2–1.4 S, and RecR_4_O_2_ complex species at ∼2.5 S. Simulations show that all of RecO is bound for RecR $ \ge$ 4.5 μM as in experiments and the area of free RecR species increase with increasing [RecR]. The RecOR complex peak is positioned at 2.5 S, reflecting that primarily RecR_4_O_2_ complex is present in a mixture of RecR_4_O and RecR_4_O_2_ complexes. The relative increase and decrease of areas of each species are similar to those in experiments, but the absolute areas differ, which is likely due to the less precise extinction coefficients at 230 nm than those at 280 nm, and we consider these differences in peak areas to be minor. (**D**) In the presence of P15, simulated data with *K*_3_ = 2.5 × 10^8^ and *K*_4_ = 1 × 10^7^ M^−1^ describe the experimental data best.

A previous study using sucrose gradient sedimentation (49) concluded that RecR is a dimer and that two RecO molecules can bind to a dimer to form a RecR_2_O_2_ complex. Our finding that RecR exists in a dimer-tetramer equilibrium prompted us to examine whether RecO can bind to a RecR dimer or a RecR tetramer or both. We first examined this in buffer BTP (pH 8.0), 25°C by titrating RecO (1.5 μM) with increasing concentrations of RecR in a series of sedimentation velocity experiments. At low concentrations of 1.5 μM and 3 μM RecR (monomer) (molar ratios of 1:1 and 1:2, Figure 4A, red and orange), a *c*(*s*) peak at ∼0.8 S is observed, reflecting unbound RecO, along with a broader *c*(*s*) peak centered at ∼2.5 S, consistent with a RecOR complex species since it has a higher sedimentation coefficient than unbound RecO (0.8 S), RecR dimer (1.2 S) or RecR tetramer (1.7 S). At RecR concentrations ≥4.5 μM (molar ratio 1:3, Figure 4A, green), the *c*(*s*) peak at ∼2.5 S increases in amplitude and a new *c*(*s*) peak at ∼1.2 S appears, corresponding to free RecR dimer. Importantly, no *c*(*s*) peak corresponding to free RecR tetramer is observed even at the highest RecR concentration of 9 μM (molar ratio 1:6, Figure 4A, gray). These observations indicate that RecO binding to RecR promotes RecR tetramerization. We also note that no *c*(*s*) peak corresponding to unbound RecO (0.8 S) is observed at RecR concentrations ≥4.5 μM, indicating all RecO is bound to RecR. Together with the observation of an unbound RecR_2_ peak (1.2 S), saturation of RecO indicates that there exists a mixture of RecOR complexes with different stoichiometries. In fact, the RecOR peak at 9 μM RecR (molar ratio of 1:6, Figure 4A, gray) shows a slight shift to the left (lower sedimentation coefficient), compared to the RecOR peak at 1.5 μM RecR (molar ratio of 1:1, Figure 4A, red). A gradual left shift of the RecOR is also observed for intermediate concentrations of RecR (Figure 4A, orange, green, blue). This suggests that upon addition of excess RecR, formation of a second RecOR complex of lower MW(RecR_4_O) occurs, due to a redistribution of RecO among the RecR molecules. This result is further discussed below along with results from sedimentation equilibrium experiments.

**Figure 5.**
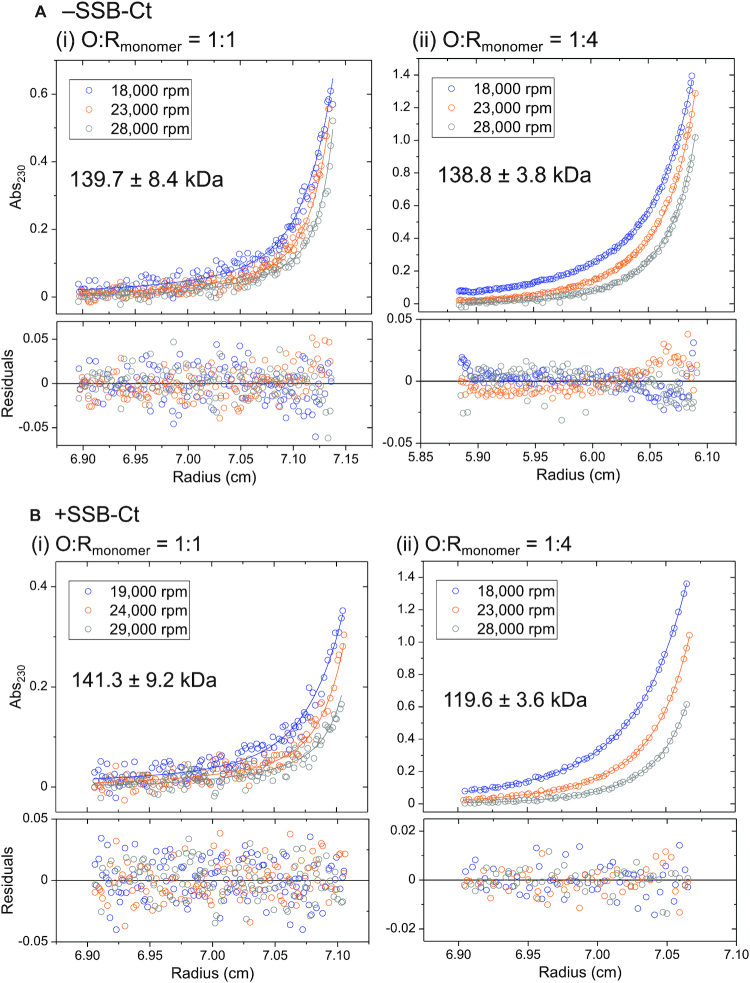
RecR tetramer can bind one or two molecules of RecO. (**A**) Results of sedimentation equilibrium experiments (monitored at 230 nm) with RecO (1.5 μM) and RecR at [RecO]:[RecR] molar ratios of 1:1 (1.5 μM RecR (monomer)) and 1:4 (6 μM RecR (monomer)) in buffer BTP (pH 8), 25°C at three rotor speeds (18 000 (blue), 23 000 (orange) and 28 000 (gray) rpm) are shown. (i) At 1:1 molar ratio, the data were described by two exponentials fit to a two species model (Equation (2)) with mass constraint where the MW of one species was fixed as that of free RecO (27.4 kDa) as observed by sedimentation velocity *c*(*s*) profile in (Figure 4A, red)). A global NLLS analysis of the data yields a MW of the second species as 139.7 ± 8.4 kDa, consistent with a RecR_4_O_2_ complex (expected MW of 142.6 kDa). (ii) At 1:4 molar ratio, the data were fit to the same model with the MW of one species fixed as that of free RecR dimer (43.9 kDa) as observed in Figure 4A (blue). A global NLLS analysis of the data yields a MW of the second species as 138.8 ± 3.8 kDa, also suggesting a RecR_4_O_2_ complex, indicating that the primary RecOR complex species is RecR_4_O_2_. The bottom panels show residuals from the fit. (**B**) Results of sedimentation equilibrium experiments (monitored at 230 nm) of RecO (1.5 μM) and RecR (1.5 μM, molar ratio 1:1) and RecR (6 μM, molar ratio 1:4) at three rotor speeds (19 000 (blue), 24 000 (orange) and 29 000 (gray) rpm) in the presence of P15 (9 μM). (i) At 1:1 molar ratio, the data were described by two exponentials and fit to a two species model with mass constraint (Equation (2)). The MW of one of the species was fixed as that of free RecO bound to P15 (29.1 kDa) as observed by sedimentation velocity *c*(*s*) profile in (Figure 4B, red)). The estimated MW of the second species is 141.3 ± 9.2 kDa, consistent with a RecR_4_O_2_ complex bound to two P15 molecules (expected MW of 146.0 kDa). (ii) At 1:4 molar ratio, the data were described by one exponential and fit to a one species model with mass constraint. The estimated MW of the second species is 119.6 ± 3.6 kDa, consistent with a RecR_4_O complex. This is in stark contrast to in the absence of P15 where the primarily observed RecOR complex species was RecR_4_O_2_.

Sedimentation equilibrium experiments were performed at two RecO to RecR molar ratios to determine the stoichiometries of the RecOR complex species. At [RecO]:[RecR]_monomer_ = 1:1 (Figure 5Ai), the data can be described by two exponentials. We therefore fit these data to a two species model where the molecular weight of one species was fixed to that of free RecO (27.4 kDa) since this species was observed in the sedimentation velocity experiment (at 0.8 S) (Figure 4Ai, red). The molecular weight of the second species was then floated and determined to be 139.7 ± 8.4 kDa from NLLS analysis of the data, consistent with the MW of a RecR_4_O_2_ complex (142.6 kDa), rather than a RecR_4_O complex (115.2 kDa).

At a higher RecR concentration, [RecO]:[RecR] = 1:4, the sedimentation equilibrium data were also well described by a two exponential fit. The sedimentation velocity data under these conditions (Figure 4A, blue) showed the presence of free RecR dimer (at ∼1.2 S). We therefore fit the data to a two species model, constraining one species to have the molecular weight of free RecR dimer (43.9 kDa). The molecular weight of the second species was then floated and determined to be 138.8 ± 3.8 kDa from NLLS analysis, also consistent with that expected for a RecR_4_O_2_ complex (142.6 kDa) (Figure 5Aii).

We considered the possibility that the RecOR species consists of a mixture of RecR_4_O_2_ and RecR_4_O species and tried fitting the sedimentation equilibrium data to a three species model while fixing the molecular weight of one species to that of RecR_2_ and floating the molecular weights of the other species. However, we could not resolve the MW of the two species using this model. We conclude from these results that the RecR_4_O_2_ species is the primary RecOR species formed at a molar ratio of 1:4. However, recall that we noted that the RecOR *c*(*s*) peak undergoes a slight shift to lower *s* values at higher RecR concentrations (Figure 4A). This suggested that RecO molecules might redistribute among RecR molecules to form a small amount of RecR_4_O complex at the large molar excess of RecR over RecO. At a higher [RecR] of 9 μM [RecO]:[RecR] = 1:6, Figure 4A, gray), we observe increases in the area of both the RecR_2_ and the RecOR peaks. Since RecO is saturated, both the increase in the area and the left shift of the RecOR peak are consistent with the formation of more RecR_4_O. For a homogeneous solution of RecR_4_O species, we expect the RecOR peak to yield a further shift left to a sedimentation coefficient of 1.9 based on the expected MW of RecR_4_O (115.2 kDa) (97).

To obtain a more quantitative interpretation of the RecO–RecR sedimentation velocity profiles in Figure 4, we used SedAnal (98) to simulate the sedimentation velocity experiments with RecO (1.5 μM) and RecR (1.5–9 μM monomers) based on Scheme 1 as described in Materials and Methods for a range of equilibrium constants. Scheme 1 describes the binding of two RecO molecules to RecR that exists in a dimer-tetramer equilibrium. *L*_obs_ is the RecR dimer–tetramer equilibrium constant determined independently from sedimentation equilibrium experiments of RecR alone. *K*_2_ is the association equilibrium constant of RecO binding to RecR dimer to form RecR_2_O. *K*_2_ was fixed at 10 M^−1^ since RecR_2_O was not observed in our experiments as discussed in the next section. *K*_3_ and *K*_4_ are the association equilibrium constants for RecO binding to RecR_4_ and RecR_4_O, respectively. The simulated data were then analyzed to obtain *c*(*s*) distribution profiles (95). The best simulated *c*(*s*) distribution profiles from the SedAnal analysis are shown in Figure 4C. The values of *K*_3_ and *K*_4_ determined from the SedAnal simulations that best describe the experimental data are *K*_3_ = 5 × 10^5^ M^−1^, *K*_4_ = 4 × 10^8^ M^−1^ (Figure 4C). These *K*_3_ and *K*_4_ values indicate that the second RecO molecule binds with higher affinity to RecR_4_ than the first RecO molecule (*K*_4_/*K*_3_ = 800), hence RecO binds with positive cooperativity to the RecR tetramer. The statistical factors associated with these equilibria are noted in Scheme 1 (Materials and Methods). Although the simulated *c*(*s*) distribution profiles in Figure 4C do not precisely reproduce the experimental profiles in Figure 4A, they capture the peak positions and differ mainly in the peak areas, which will be greatly affected by the accuracy of the extinction coefficients that were used in the analysis. Since the extinction coefficients at 230 nm are less precise than those at 280 nm, we consider these differences in peak areas to be minor.

### RecR_2_O is not populated at equilibrium

We observed that RecOR complexes form only with RecR tetramers at equilibrium, whereas no complexes were observed with RecR dimers. The predicted molecular weight of a RecR_2_O complex is 71.3 kDa, and we would expect the RecR_2_O species to be observed in a sedimentation velocity experiment at ∼1.4 S, as estimated from its molecular weight. Although this value is closer to the sedimentation coefficient of RecR dimer at 1.2 S and would be difficult to resolve, a significant peak at 1.4 S is not observed (Figure 4A) (110). Furthermore, the expected MW does not correspond to either of the estimated molecular weights from our sedimentation equilibrium experiments (Figure 5Ai and ii). Two RecO molecules bound to a RecR dimer would have a MW of 98.7 kDa, which also does not correspond to the estimated molecular weights in Figure 5Ai and ii.

**Figure 6.**
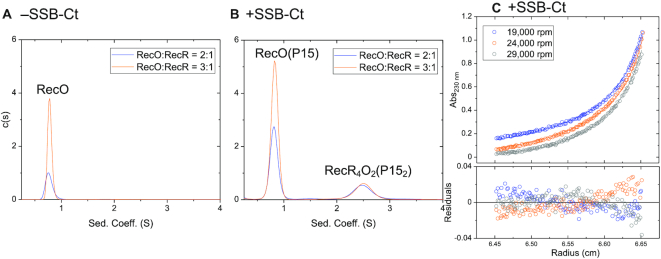
Binding of the SSB Ct acidic tip to RecO enhances its affinity for the RecR tetramer. Sedimentation velocity (monitored at 230 nm) *c*(*s*) distribution profiles in buffer BTP (pH 8.0) at 25°C titrating RecR (2 μM in monomers) with RecO (4 μM (blue) and 6 μM (orange)). (**A**) In the absence of P15, only free RecO is observed at 0.8 S and no RecOR complex species is observed. However, a decrease in the initial absorbance compared to an expected value predicted from initial [RecO] and [RecR] indicates that larger RecOR complex aggregate has formed and sedimented to the bottom of the cell. (**B**) In the presence of P15, (24 μM for 2:1 molar ratio and 36 μM for 3:1 molar ratio, 6-fold molar excess to RecO), a RecOR complex is observed at ∼2.5 S along with free RecO at 0.8 S at the two molar ratios of RecO to RecR. Increasing [RecO] from 2:1 molar ratio to 3:1 increases the area of the free RecO peak (0.8 S) but does not increase the area of the RecOR complex peak, indicating RecR is saturated with RecO. (**C**) Results of sedimentation equilibrium experiments (monitored at 230 nm) performed in buffer BTP (pH 8.0) at 25°C with RecR (2 μM_monomer_), RecO (6 μM, 3:1 molar ratio) and P15 (36 μM, P15/RecO molar ratio of 6:1) at three rotor speeds (19 000 (blue), 24 000 (orange) and 29 000 (gray) rpm). The data were described by two exponentials and fit to a two-species model with mass constraint (Equation (2)) with the MW of one species fixed at 29.1 kDa (RecO–P15 complex). A global NLLS analysis of the data yielded a MW for the second species of 144.5 ± 3.1 kDa, suggesting a RecR_4_O_2_ complex bound with two P15 peptides.

To further examine the possibility that a RecR_2_O complex can form, sedimentation velocity experiments were performed with increasing excess concentrations of RecO added to a constant concentration of RecR (2 μM (monomer)) (Figure 6A). When RecO is in excess over RecR, only one species is observed at 0.8 S, corresponding to free RecO, with no evidence of any RecR or RecOR species (Figure 6A). However, under these conditions we noted a decrease in the initial absorbance in the cell compared to what was expected based on the initial total concentrations of RecO and RecR proteins in the sample. This indicates that a larger RecOR complex (aggregate) had formed that sedimented to the bottom of the cell. However, we found no evidence for a soluble RecR_2_O species.

### Binding of the SSB C-terminal acidic tip to RecO stabilizes a RecR_4_O complex

We next examined whether the binding of the *E. coli* SSB C-terminal acidic tip to RecO has any effect on RecO-RecR interactions. Our previous studies showed that a 15-residue peptide containing the C-terminal tip of SSB (PSNEPPMDFDDDIPF), referred to as P15, forms a 1:1 complex with RecO with equilibrium constant *K* = (1.2 ± 0.3) × 10^7^ M^−1^ in buffer BTP (pH 8.0) at 25°C (26). Based on this binding affinity, a 6-fold molar excess of P15 over RecO should result in 98.8% of RecO bound with P15 at 1.5 μM RecO and 99.2% at 2 μM RecO. We therefore performed the following experiments with a six-fold molar excess of P15 over the [RecO].

Sedimentation velocity experiments were performed at a constant RecR concentration (2 μM (monomer)) and multiple excess RecO concentrations in the presence of P15. In contrast to the results in the absence of P15 (Figure 6A), a RecOR complex species is observed at 2.5 S (Figure 6B) in the presence of excess RecO and P15. Sedimentation equilibrium experiments were then performed at a 3:1 [RecO]:[RecR] molar ratio (6 μM RecO, 2 μM RecR) in the presence of P15 (36 μM) to estimate the molecular weight of the RecOR species at 2.5 S. The data in Figure 6C are well described by a two exponential fit indicating the presence of two major species. We analyzed these data using a two-species model with mass constraint, fixing the MW of one species at 29.1 kDa, corresponding to the RecO–P15 species evident in Figure 6B and floating the MW of the larger species. This yielded a molecular weight estimate of 144.5 ± 3.1 kDa for the larger species, consistent with a RecR_4_O_2_ complex (146.0 kDa) bound with two P15 molecules (Figure 6C) indicating that a RecR_4_O_2_ complex can form in the presence of P15. Recall that in the presence of excess RecO over RecR in the absence of P15, large RecOR complexes form that sediment to the cell bottom in a sedimentation velocity experiment, which is why they are not observed in Figure 6A.

We next performed sedimentation velocity experiments with RecO (1.5 μM) and increasing concentrations of RecR in the presence of a 6-fold excess of P15 over RecO. The species distributions (Figure 4B) show free RecO and some dimeric RecR at <1.5 S, and larger species between 2.2 and 2.5 S. At a molar ratio of 1:1 (Figure 4B, red), RecOR complexes are observed at ∼2.5 S along with free RecO at 0.8 S. The position of the *c*(*s*) peak for the RecOR complexes shows a notable shift from 2.5 to 2.2 S with increasing [RecR] suggesting formation of a smaller RecOR complex. At the higher RecR/RecO molar ratio of 4:1 (Figure 4B, blue), a RecOR complex is observed at ∼2.3 S with no free RecO or RecR_2_ evident. This is in clear contrast to what is observed in the absence of P15 where free RecR dimer is observed (Figure 4A, blue). This suggests that all of the RecR is bound to RecO at a 4:1 RecR/RecO molar ratio in the presence of P15 indicating that P15 promotes RecR_4_O complex formation.

To obtain a more quantitative determination of the effect of P15 on the formation of RecR_4_O versus RecR_4_O_2_, we performed sedimentation equilibrium experiments in the presence of P15 at different [RecO]:[RecR] molar ratios (Figure 5B). At a 1:1 ratio, the data are well described by a two-species model (Figure 5Bi). By constraining the MW of one species to be that of free RecO, we estimate the MW of the second species to be 141.3 ± 9.2 kDa, consistent with a RecR_4_O_2_ complex, similar to what is observed in the absence of P15 (Figure 5Ai). However, at a 1:4 molar ratio (Figure 5Bii), the data are well described by a single exponential indicating a single species, consistent with the sedimentation velocity results at this RecO/RecR ratio (Figure 4B, blue). A fit of the sedimentation equilibrium data in Figure 5Bii to a one-species model yields a molecular weight of 119.6 ± 3.6 kDa, consistent with a RecR_4_O complex. These results show that both RecR_4_O and RecR_4_O_2_ complexes are able to form both in the absence and the presence of P15, however P15 binding shifts the equilibrium to favor the RecR_4_O species. This interpretation is consistent with the shift in the peak of the *c*(*s*) distribution for the RecOR complex from ∼2.5 to ∼2.2 S with increasing [RecR] (Figure 4B).

To obtain a more quantitative interpretation of these sedimentation velocity experiments we used SedAnal (98) to simulate the sedimentation velocity profiles for the experiments performed with RecO (1.5 μM) and RecR (1.5–9 μM monomers) based on Scheme 1 (see Materials and Methods) and these simulated profiles were then analyzed by Sedfit (95) to obtain c(s) distribution profiles. These simulated *c*(*s*) profiles are shown in Figure 4D. The parameters that describe the data best in the presence of P15 are *K*_3_ = 2.5 × 10^8^ M^−1^ and *K*_4_ = 1 × 10^7^ M^−1^, which differ considerably from the values estimated in the absence of P15. Comparison of the values of *K*_3_ and *K*_4_ estimated in the presence and absence of P15 shows a dramatic effect of P15 on both equilibrium constants. In the presence of P15, the value of *K*_3_ increases whereas the value of *K*_4_ decreases compared to their values in the absence of P15. This results in a much lower ratio of *K*_4_/*K*_3_ = 4 × 10^−2^ in the presence of P15 indicating negative cooperativity. Hence, in the presence of P15, the first RecO molecule now binds stronger and the second RecO binds weaker to RecR_4_. Recall that in the absence of P15 RecO binding to the RecR tetramer displays positive cooperativity.

**Figure 7.**
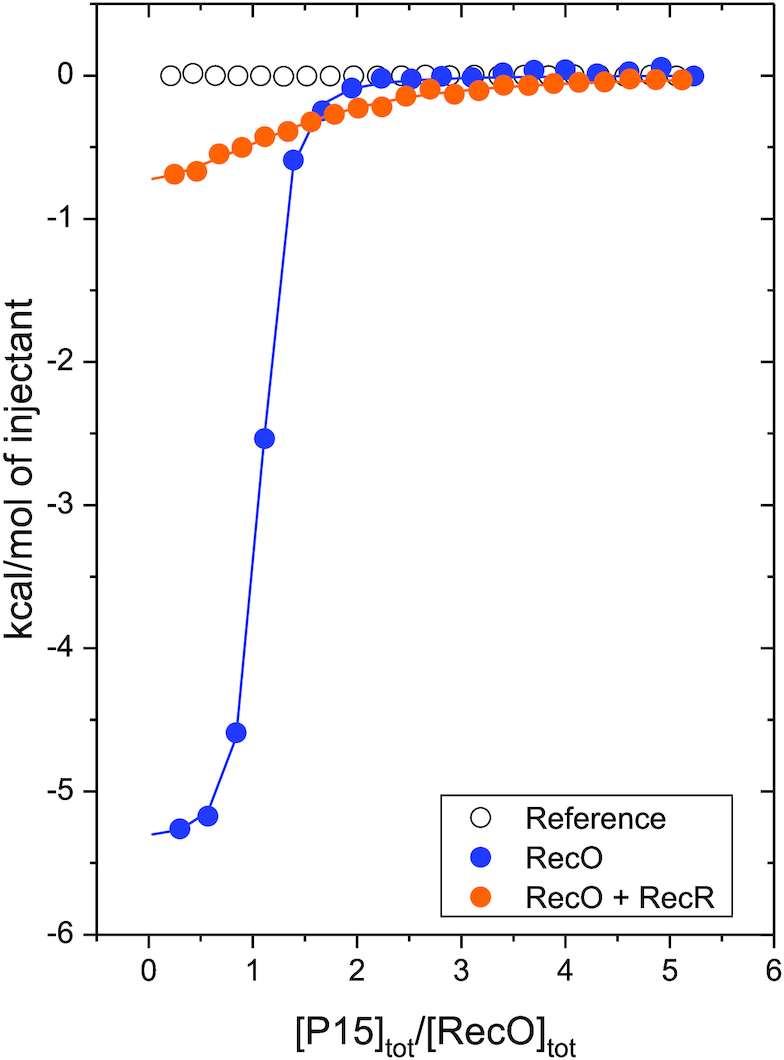
RecR reduces the affinity of RecO for the SSB Ct acidic tip peptide. Results of ITC titrations (buffer BTP, pH 8 at 25°C) of RecO (2 μM) with P15 peptide fit to a 1:1 binding model with equilibrium binding constant of (1.2 ± 0.3) × 10^7^ M^−1^ and Δ*H* = –5.2 ± 0.1 kcal/mol (reproduced from (26), blue), and a mixture of RecO (2 μM) and RecR (8 μM monomer) (1:4 molar ratio) with P15 (orange). In the presence of RecR, the RecO–P15 binding enthalpy, Δ*H*, is significantly reduced to less than 1 kcal/mol with lower apparent binding affinity. A reference titration of RecR with P15 (open circles) indicates no binding of P15 to RecR.

We next performed an ITC experiment to examine the effect of RecR on P15 binding to RecO. P15 was titrated into buffer BTP (pH 8.0) at 25°C containing a 1:4 molar ratio of RecO to RecR (monomer), with RecO at 2 μM, the same concentration used in a previous study of P15 binding to RecO (26). The reported association equilibrium constant for the P15–RecO interaction is *K*_O-P15_ = (1.2 ± 0.3) × 10^7^ M^−1^ with Δ*H* = –5.2 ± 0.1 kcal/mol. In the presence of RecR, the measured Δ*H* for the P15–RecO interaction is less than 1 kcal/mol, near the limit of detection of the instrument, and therefore we are unable to obtain accurate binding parameters. Qualitatively, however, both the binding affinity and enthalpy are clearly reduced significantly in the presence of RecR (Figure 7, orange). Since RecR does not interact with P15 (Figure 7, empty circles), the excess RecR species does not contribute to the measured enthalpy change. These results show that RecR binding to RecO lowers the RecO binding affinity for P15, consistent with the observation that P15 binding to RecO lowers the affinity of RecO for RecR to stabilize RecR_4_O complex over RecR_4_O_2_.

## DISCUSSION

*Escherichia coli* RecO is an essential recombination mediator protein in the RecF DNA repair pathway, which also involves RecF and RecR along with RecQ helicase, RecJ exonuclease, and SSB (48,111). It has been shown that RecOR, without RecF, can function to load RecA onto ssDNA (21). *Escherichia coli* RecR, despite its lack of DNA binding activity, is required for RecO to initiate homologous recombination. Although previous studies have characterized RecO and RecR proteins from other organisms in detail, the assembly states, stoichiometries and binding energetics of *E. coli* RecOR complexes were still unclear. Important questions include which species of RecO, RecR and RecOR complexes are functional in HR initiation and how these species interact with ssDNA-bound SSB to be recruited to the site of DNA damage. When a RecOR complex is bound by SSB, SSB must then dissociate from ssDNA and be replaced by the RMP (22,112–114).

### RecO binds preferentially to the RecR tetramer at equilibrium

A previous report using sucrose gradient sedimentation analysis and a single concentration of RecR (0.2 μM) under solution conditions (35 mM Tris–HCl, pH 7.5, 10 mM MgCl_2_ and 1.8 mM dithiothreitol, 4°C) that differ from those used in our study concluded that concluded that *E. coli* RecO is monomeric and *E. coli* RecR is dimeric (60). While our studies support the conclusion that RecO is a stable monomer, we find that RecR exists in a dimer–tetramer equilibrium that is pH-dependent, with lower pH stabilizing tetramers. Our studies were performed over a range of pH values and salt conditions (50 or 200 mM NaCl in the presence and absence of 10 mM MgCl_2_) for a range of RecR concentrations (2–20 μM) in the presence of 25% glycerol to enhance protein solubility. The formation of tetramers is accompanied by an uptake of protons in the pH range from 8.0 to 9.0. We observe that inclusion of 10 mM MgCl_2_ promotes RecR dimerization, which may explain why RecR tetramers were not observed in the previous study (59), although differences in the solution conditions of the two studies (e.g. sucrose versus glycerol) are also likely to affect the dimer–tetramer equilibrium.

A crystal structure of the *D. Radiodurans* RecR tetramer (87) shows interactions between subunits via both the N- and C-termini to form a ring-like structure (Figure 1b), which may encircle and bind to DNA as a clamp (84). Furthermore, a deletion mutant of *D. radiodurans* RecR lacking the N-terminal HhH motif exists as a stable dimer in solution, and stable *D. radiodurans* RecR dimers have also been reported to form by N-termini interactions (85). An NMR study of *T. thermophilus* RecR also shows that dimerization occurs at the N-terminal interface (87,89). The *E. coli* RecR does not interact with DNA, unlike *D. radiodurans and T. thermophilus*, yet RecO requires RecR to initiate homologous recombination (83,87,115). The fact that RecR exists in a dimer-tetramer equilibrium may be important for loading of a RecR tetramer onto ssDNA, especially if the tetramer encircles the ssDNA as has been suggested for *D. radiodurans*.

An estimate of the RecR monomer concentration *in vivo* ranges from ∼50 nM to 250 nM, depending on growth conditions (116). The lowest concentration examined in our study was 2 μM_monomer_, which shows a larger fraction of dimers than tetramers. Yet, we observe that only RecR tetramers are bound to RecO to form both RecR_4_O and RecR_4_O_2_ complexes at equilibrium. Hence, RecO promotes tetramerization of RecR. Similarly, the presence of *D. radiodurans* RecO has also been reported to promote *D. radiodurans* RecR tetramerization (85).

The estimated concentration of RecO *in vivo* is ∼30 to 40 nM, 1.5- to 62-fold less than the estimated RecR concentration *in vivo* (116). At these concentrations, we expect both RecR_4_O and RecR_4_O_2_ complexes to co-exist. Hence, it is unclear whether only one or both forms of the RecOR complex function to initiate RecA loading. Of course, these species distributions will likely be affected by DNA binding, although we have not examined these effects in this study. However, only RecO has been shown to bind ssDNA (22,83). A proposed model for RecOR complex loading RecA protein onto ssDNA is described below.

Scheme 1 (see Materials and Methods) represents the complete equilibrium binding scheme that applies to the RecO/RecR system. However, at equilibrium, we find no evidence for RecR_2_O complexes under our conditions at equilibrium, hence the dominant species at equilibrium are shown in the red box in Scheme 1. However, this does not eliminate the possibility that a RecR_2_O species may be an important transient intermediate in the pathway for formation of the RecR_4_O and RecR_4_O_2_ complexes. In fact, if a RecR tetramer functions as a clamp around the ssDNA, this would likely occur via RecO binding to a RecR dimer followed by formation of the tetramer around the ssDNA. Evidence for such a pathway must await transient kinetic studies of the assembly of RecOR complexes.

### The SSB-Ct has an allosteric effect on RecOR complex formation

*Escherichia coli* SSB protein forms a stable tetramer composed of subunits (177 amino acids per monomer) that are composed of two domains, an N-terminal 112 amino acid DNA binding domain, which forms an OB-fold, and a 65 amino acid intrinsically disordered C-terminal domain, the last 9 amino acids of which, the Ct acidic tip, form the major site of interaction with an array of genome maintenance proteins (SIPs), including RecO. The 56 amino acid intrinsically disordered linker (IDL) is essential for cooperative binding of SSB to ssDNA (96,117,118). RecO can bind the SSB-Ct acid tip, such that up to four RecO molecules can bind to one SSB tetramer at its four C-terminal tips (26). The general view has been that the binding of SIPs to the Ct acidic tip of SSB mainly provides a mechanism to tether the SIP to SSB in order to sequester it near its site of interaction on the DNA. However, we show here that the Ct acidic tip also serves as an allosteric effector of RecO interactions with RecR. We show that at a 4:1 molar ratio of RecR/RecO in the absence of SSB-Ct, RecO binds to a RecR tetramer primarily with a stoichiometry of two RecO molecules per tetramer. However, upon addition of an SSB-Ct peptide, at the same 4:1 molar ratio of RecR/RecO, RecR_4_O is observed to be the primary species. Hence binding of the SSB-Ct to RecO shifts the RecR_4_O–RecR_4_O_2_ equilibrium to favor RecR_4_O.

A crystal structure of *E. coli* RecO shows the SSB-Ct tip bound in a hydrophobic pocket in the central alpha helical region (Figure 1), separate from the RecR binding interface that is localized on the N-terminal DNA binding domain of RecO (90) indicating that the effect of the SSB-Ct on RecO binding to RecR is allosteric. Allosteric effects of the SSB-Ct acidic tip on the properties of other SIPs, such as *E. coli* RecQ helicase and RadD, have also recently been demonstrated (61,80,119). The binding of just the Ct acidic tip to RecQ has been shown to stimulate its DNA unwinding activity (61) and the ATPase activity of *E. coli* RadD is stimulated by SSB-Ct peptide both in the presence and absence of DNA (80). Hence, the SSB C-terminal tail should be viewed as potentially serving as more than simply a tether, since it can also modify the properties of at least three SIPs. It will be of great interest to see whether this effect is observed for other SIPs.

### A model for RecOR loading of RecA protein onto ssDNA

**Figure 8.**
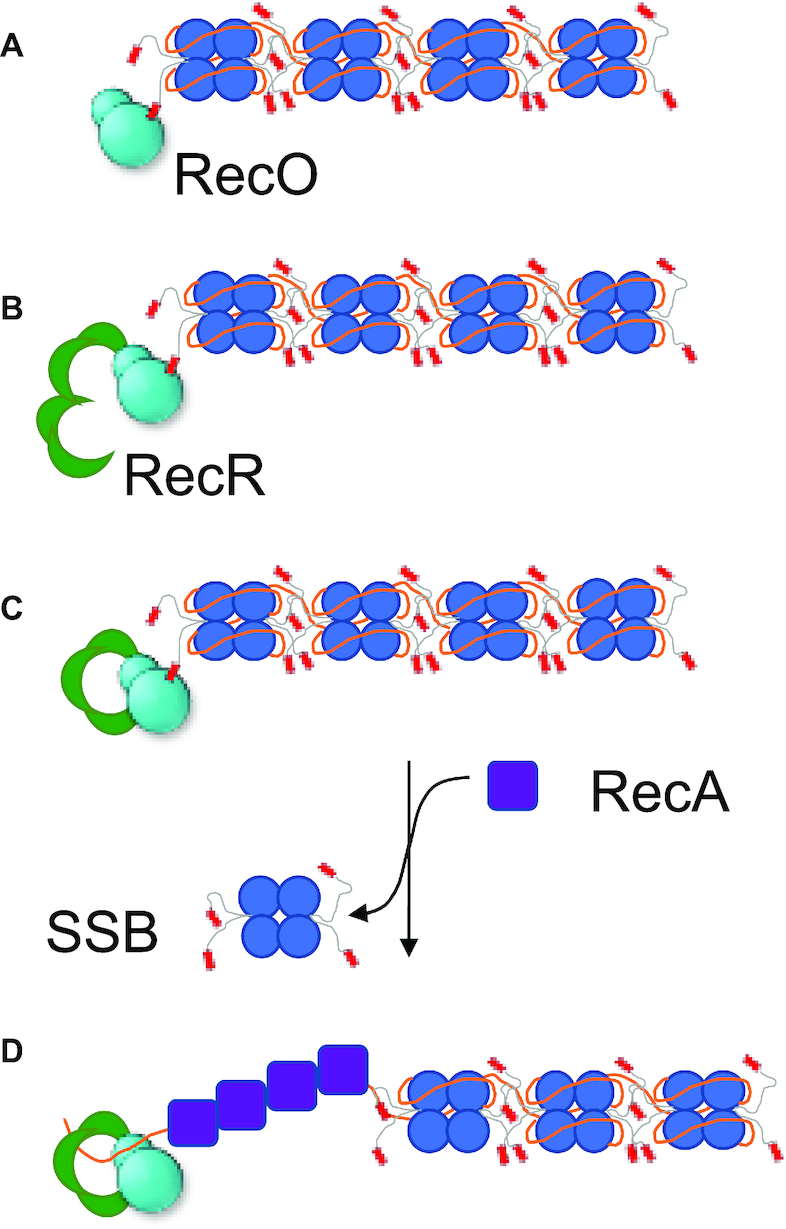
A proposed mechanism for RecOR loading of RecA protein onto ssDNA. Our results suggest a proposed mechanism for RecOR loading of RecA onto ssDNA (solid orange line) that is coated with SSB. (**A**) In this model, the SSB, while bound to ssDNA, recruits RecO via the C-terminal tip (SSB-Ct). (**B**) RecR, in a dimer-tetramer equilibrium, binds to RecO, which promotes tetramerization of RecR. (**C**) SSB-Ct bound RecO forms a complex with RecR, favoring the formation of a RecR_4_O complex over a RecR_4_O_2_. The absence of the second RecO may facilitate the RecR_4_ tetramer to recruit RecA to be loaded onto the ssDNA gap for repair. (**D**) SSB-Ct dissociates from RecR_4_O complex, and SSB is displaced as RecA is loaded onto ssDNA.

Based on the results reported here, we suggest a model for the RecA loading pathway facilitated by RecOR as depicted in Figure 8. In this model, a single strand gap is first coated with tightly bound SSB protein. Then, one (or more) RecO is bound to SSB via its C-terminal acidic tip which facilitates RecO binding to ssDNA (Figure 8A). RecO then binds to RecR to form a RecOR complex on ssDNA, favoring the formation of a RecR_4_O complex, with a RecR tetramer encircling the DNA (Figure 8B and C). As discussed above, this pathway could involve an intermediate in which RecO binds transiently to a RecR_2_ dimer. After formation of a RecR_4_O_2_, one of the RecO molecules would likely dissociate due to negative cooperativity that stabilizes a RecR_4_O complex. The absence of the second RecO may allow the RecR_4_ tetramer to recruit RecA to be loaded onto the ssDNA gap. The ensuing formation of a RecA filament on the ssDNA results in SSB displacement (Figure 8D). Previous studies suggest that the RecOR pathway for loading RecA is more efficient for uninterrupted lengths of SSB-coated DNA whereas a separate RecFOR pathway is more efficient near duplex regions (21). Although binding of RecO in the presence and in the absence of RecR has been studied with a short ssDNA molecule, (dT)_15_ (22), the binding properties of RecO and RecOR complex to longer ssDNA are less clear. Quantitative studies of the binding of RecO and RecOR to DNA and the effect of SSB-Ct on those binding properties should inform a better understanding of the RecOR pathway.

## Supplementary Material

gkaa1291_Supplemental_File
