# Supplementary material for: Allosteric effects of SSB C-terminal tail on assembly of E. coli RecOR proteins
Source: Nucleic Acids Res. 2021 Jan 15;49(4):1987–2004. doi: 10.1093/nar/gkaa1291 (PMC7913777; doi:10.1093/nar/gkaa1291)
Supplement: gkaa1291_Supplemental_File [file gkaa1291_supplemental_file.pdf]

SUPPLEMENTARY MATERIAL

Allosteric Effects of SSB C-terminal Tail on Assembly of *E. coli* RecOR Proteins

Min Kyung Shinn[1,2], Alexander G. Kozlov[1] and Timothy M. Lohman[1,*]

[1]Department of Biochemistry and Molecular Biophysics, Washington University in St. Louis School of Medicine, St. Louis, MO 63110

[2]Department of Physics, Washington University in St. Louis, St. Louis, MO 63130

*To whom correspondence should be addressed. Tel: +1 314 362 4393; Fax +1 314 362 7183; Email: lohman@wustl.edu

Potential effects of glycerol on the sedimentation studies

Buffer BTP used in our experiments contains 25%(v/v) glycerol which increases the solubility of the proteins and thus concerns can be raised about whether a glycerol gradient that can form may affect the conclusions drawn from the sedimentation experiments. Although a glycerol gradient will form in these experiments, its effect does not influence our conclusions as discussed in several recent studies of other interacting systems (1,2). First of all, we only use the results of the sedimentation velocity (SV) studies to determine whether higher order RecOR complexes can form. We do not use the SV results to determine the molecular weights of the higher order complexes. Rather, we use the sedimentation equilibrium studies to determine these molecular weights, and thus the RecOR stoichiometries. Nevertheless, following the suggestion of Gabrielson et al., we also analyzed the SV data by truncating the data at ~6.7 cm, which eliminates the data that is most affected by any glycerol gradient (2,3). Analysis of the truncated data did not affect the results significantly, as we find that the sedimentation coefficients differ by < 2% for the c(s) distributions in Figures 2, 3a, 4a, 4b, 6a, and 6b.

The sedimentation equilibrium (SE) experiments were performed at lower rotor speeds than the SV experiments hence any glycerol gradient should be even smaller in these experiments. The global fits of all of the data for RecR assembly (3 concentrations and 3 speeds at each pH) shown in Figure S3 show good residuals using the partial specific volume determined for RecR in BTP buffer (25% glycerol) and the density for BTP buffer (25% glycerol) indicating that there is no need to correct for any glycerol gradient in those experiments as well as the RecR-RecO SE experiments. The main consideration for the RecR-RecO SE experiments is whether a glycerol gradient would influence our determinations of the molecular weights of these complexes. Put another way, the question is how different would the product of partial specific volume ($\bar{\upsilon}$) and solvent density (ρ) need to be to lead to uncertainty in the determination of whether the RecR tetramer is bound to one RecO or two RecO molecules, a molecular weight difference of 27.4 kDa. We first consider the potential effect of glycerol concentration on $\bar{\upsilon}$ that would be needed for such uncertainty. The $\bar{\upsilon}$ for the $RecR_4O$ complex would need to be at least 5.9% higher (0.758 ml/g) than the value of 0.716 ml/g calculated from the experimentally determined $\bar{\upsilon}$ values of RecO

and RecR in BTP buffer. This value of 0.758 ml/g is considerably outside our experimental error and much higher than $\bar{\upsilon}$ for most proteins even at high glycerol concentrations (4,5). Coleman et al. (6) state that $\bar{\upsilon}$ should increase linearly with glycerol concentration and the expected effect of an increase from 0 to 40% is only 0.01 ml/g. Hence the effect of glycerol on $\bar{\upsilon}$ is too small to influence our conclusions. The other effect of a higher glycerol concentration due to a gradient would be to increase the solvent density, ρ. In order to have an uncertainty in MW corresponding to one RecO in the $RecR_4$-RecO stoichiometry, the product, $\bar{\upsilon}\rho$ for $RecR_4O$ would need to be 0.812 compared to its value of 0.767 in buffer BTP (25% glycerol). Assuming no effect on $\bar{\upsilon}$, this would require the glycerol concentration to be ~45%(v/v) instead of 25%(v/v) at the radial position where the RecR-RecO complex equilibrates, which is much higher than expected to be produced by a glycerol gradient at the low rotor speeds used in the SE experiments (1,2).

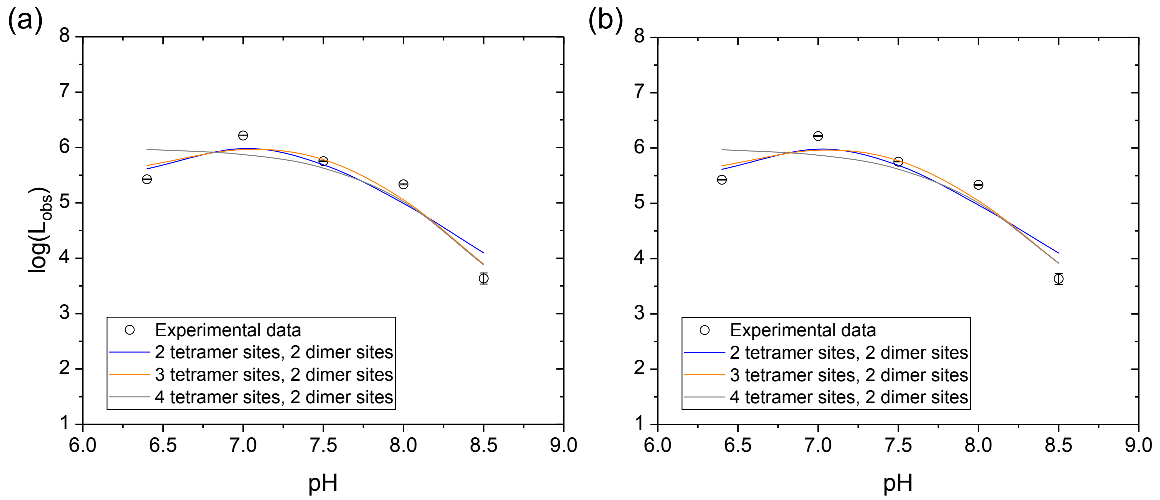

Figure S1 Plot of log(L_{obs}) vs. pH obtained from sedimentation equilibrium experiments of RecR (Fig. 3b). The experimental data from Fig. 3d are shown in empty circles. The solid lines show NLLS fits to a dimer-tetramer equilibrium model (Eqs. (6) and (7)) assuming two independent protonation sites on the RecR dimer and two (blue), three (orange), and four (gray) independent or cooperative sites on the tetramer. (a) The dataset was fitted to two independent dimer sites and two (blue), three (orange), and four (gray) independent sites on the tetramer. The R^2 values for fitting to each model are 0.96, 0.98, and 0.96, respectively. The fit to three independent protonation sites on the tetramer (solid orange line) describes data the best. (b) The dataset was fitted to two independent dimer sites and two (blue), three (orange), and four (gray) cooperative sites on the tetramer. The R^2 values for fitting to each model are 0.97, 0.99, and 0.96, respectively. The three cooperative protonation sites on the tetramer (solid orange line) describes the data best. We chose the latter case, showing the best R^2 value as the most adequate model describing the data and present this fitting in Fig. 3d.

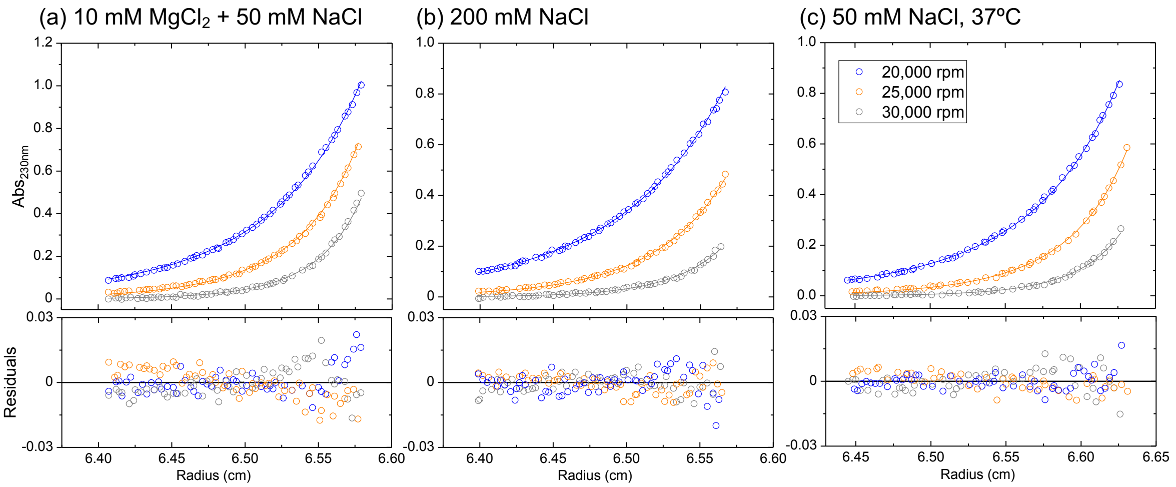

Figure S2 Sedimentation equilibrium (monitored at 230 nm) of 8 μM *E. coli* RecR monomer at the three rotor speeds (20,000 (blue), 25,000 (orange), and 30,000 (gray) rpm) (a) in buffer BTP (pH 8.0) at 25°C with additional 10 mM $MgCl_2$ and (b) in buffer BTP with 200 mM NaCl and (c) buffer BTP at 37°C. A global NLLS fitting of experiments performed at 4, 8, and 12 μM RecR monomer to the "Dimer-Tetramer model" described in Methods estimated tetramerization equilibrium constants as $(7.20 \pm 0.83) \times 10^4$ M^{-1} and $(3.49 \pm 0.48) \times 10^4$ M^{-1}, and $(1.49 \pm 0.17) \times 10^5$ M^{-1}, as noted in Table 1. The equilibrium constants are reduced in the presence of higher [NaCl] and [$MgCl_2$] comparison to buffer BTP at 50 mM NaCl (Table 1, $(2.16 \pm 0.05) \times 10^5$ M^{-1}). Lower tetramerization equilibrium constants stabilize the dimeric form of RecR. The equilibrium constant obtained at 37°C is similar to the value at 25°C. Hence the dimer-tetramer equilibrium is notably affected by salt concentrations but not as affected by temperature.

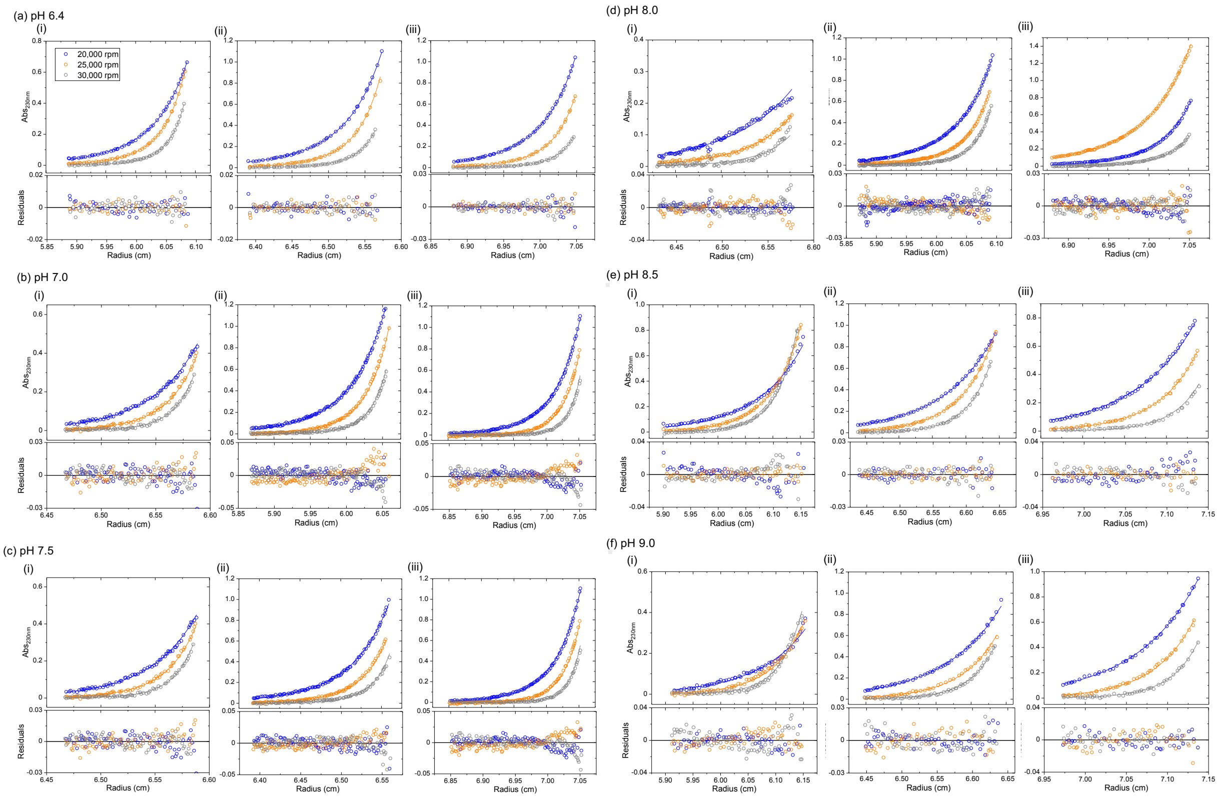

Figure S3 Global NLLS fittings of sedimentation equilibrium (monitored at 230 nm) of *E. coli* RecR at the three rotor speeds (20,000 (blue), 25,000 (orange), and 30,000 (gray) rpm) in buffer BTP (25ºC) at (a) pH 6.4 (b) pH 7.0 (c) pH 7.5 (d) pH 8.0 (e) pH 8.5 and (f) pH 9.0 at (i) 4 μM, (ii) 8 μM, and (iii) 12 μM (in monomers). The nine data sets total, three rotor speeds from the three concentrations, at each pH were globally fitted to the "dimer-tetramer model" described in Methods and estimated tetramerization equilibrium constants as noted in Table 1. All sedimentation equilibrium experiments of RecR shown in Figs. 3 and S2 are similarly fitted to the "dimer-tetramer model" by global NLLS fittings of data sets from three rotor speeds at three RecR concentrations.

pH	50 mM NaCl	200 mM NaCl	10 mM $MgCl_2$
6.4	0.0028		
7.0	0.0101		
7.5	0.0080		
8.0	0.0067	0.0089	0.0107
	0.0126 (37ºC)		
8.5	0.0088		

Table S1 RMSD values from global fittings of sedimentation equilibrium experiments of RecR at 4, 8, and 12 μM for each indicated solution condition. The tabulated RMSD values show good fits to the dimer-tetramer model since the values are near or below the level of noise in data acquisition.

References for Supplementary Material

1. Veronese, P.K. and Lucius, A.L. (2010) Effect of temperature on the self-assembly of the Escherichia coli ClpA molecular chaperone. *Biochemistry*, **49**, 9820-9829.
2. Lin, J. and Lucius, A.L. (2015) Examination of the dynamic assembly equilibrium for E. coli ClpB. *Proteins*, **83**, 2008-2024.
3. Gabrielson, J.P., Arthur, K.K., Kendrick, B.S., Randolph, T.W. and Stoner, M.R. (2009) Common excipients impair detection of protein aggregates during sedimentation velocity analytical ultracentrifugation. *J Pharm Sci*, **98**, 50-62.
4. Gekko, K. and Timasheff, S.N. (1981) Mechanism of protein stabilization by glycerol: preferential hydration in glycerol-water mixtures. *Biochemistry*, **20**, 4667-4676.
5. Cole, J.L. (1996) Characterization of Human Cytomegalovirus Protease Dimerization by Analytical Centrifugation. *Biochemistry*, **35**, 15601-15610.
6. Coleman, J., Eaton, S., Merkel, G., Skalka, A.M. and Laue, T. (1999) Characterization of the self association of Avian sarcoma virus integrase by analytical ultracentrifugation. *J Biol Chem*, **274**, 32842-32846.
